# Achieving Remission in Gulf War Illness: A Simulation-Based Approach to Treatment Design

**DOI:** 10.1371/journal.pone.0132774

**Published:** 2015-07-20

**Authors:** Travis J. A. Craddock, Ryan R. Del Rosario, Mark Rice, Joel P. Zysman, Mary Ann Fletcher, Nancy G. Klimas, Gordon Broderick

**Affiliations:** 1 Institute for Neuro Immune Medicine, Nova Southeastern University, Ft. Lauderdale, FL, United States of America; 2 Center for Psychological Studies, Nova Southeastern University, Ft. Lauderdale, FL, United States of America; 3 Graduate School for Computer and Information Sciences, Nova Southeastern University, Ft. Lauderdale, FL, United States of America; 4 College of Osteopathic Medicine, Nova Southeastern University, Ft. Lauderdale, FL, United States of America; 5 Center for Computational Science, University of Miami, Miami, FL, USA; 6 Veterans Affairs Medical Center, Miami, FL, United States of America; 7 College of Pharmacy, Nova Southeastern University, Ft. Lauderdale, FL, United States of America; University of California, San Francisco, UNITED STATES

## Abstract

Gulf War Illness (GWI) is a chronic multi-symptom disorder affecting up to one-third of the 700,000 returning veterans of the 1991 Persian Gulf War and for which there is no known cure. GWI symptoms span several of the body’s principal regulatory systems and include debilitating fatigue, severe musculoskeletal pain, cognitive and neurological problems. Using computational models, our group reported previously that GWI might be perpetuated at least in part by natural homeostatic regulation of the neuroendocrine-immune network. In this work, we attempt to harness these regulatory dynamics to identify treatment courses that might produce lasting remission. Towards this we apply a combinatorial optimization scheme to the Monte Carlo simulation of a discrete ternary logic model that represents combined hypothalamic-pituitary-adrenal (HPA), gonadal (HPG), and immune system regulation in males. In this work we found that no single intervention target allowed a robust return to normal homeostatic control. All combined interventions leading to a predicted remission involved an initial inhibition of Th1 inflammatory cytokines (Th1Cyt) followed by a subsequent inhibition of glucocorticoid receptor function (GR). These first two intervention events alone ended in stable and lasting return to the normal regulatory control in 40% of the simulated cases. Applying a second cycle of this combined treatment improved this predicted remission rate to 2 out of 3 simulated subjects (63%). These results suggest that in a complex illness such as GWI, a multi-tiered intervention strategy that formally accounts for regulatory dynamics may be required to reset neuroendocrine-immune homeostasis and support extended remission.

## Introduction

Of the approximately 700,000 veterans returning from the First Gulf War, one-third have been afflicted with a chronic multisymptom disorder known as Gulf War Illness (GWI) [1]. Characterized by severe and debilitating symptoms including fatigue, musculoskeletal pain, and cognitive problems, GWI affects multiple bodily systems, has no known cure and requires long-term treatment and monitoring [1]. While the cause and illness mechanisms of GWI are largely unknown, a leading hypothesis points to the involvement of a neuroinflammatory cascade triggered by exposure to a neurotoxin and exacerbated by the stress of a combat environment [2–4]. Such neuroinflammatory processes would be consistent with the broad range of disparate symptoms observed in GWI that extend beyond the central nervous system and affect endocrine and immune function. The hypothalamic-pituitary-adrenal (HPA) axis is major information highway linking these peripheral systems to the brain and plays a central role in the body’s response to environmental stressors [5–7]. Evidence of HPA axis dysfunction has been reported in GWI [8]. While several mathematical models of HPA dynamics exist [9–14], only one accommodates multistability in the HPA axis via the inclusion of more detailed feed-forward regulation [14]. Unfortunately this model and the majority of other models do not extend in scope beyond the HPA axis. This is a significant limitation since HPA regulatory activity is intertwined with that of the hypothalamic-pituitary-gonadal (HPG) axis and the immune system, among others. This integrated connectivity is at the heart of the body’s multi-stable adaptive behavior and is simultaneously responsible for its resilience and potential vulnerability

**Fig 1:**
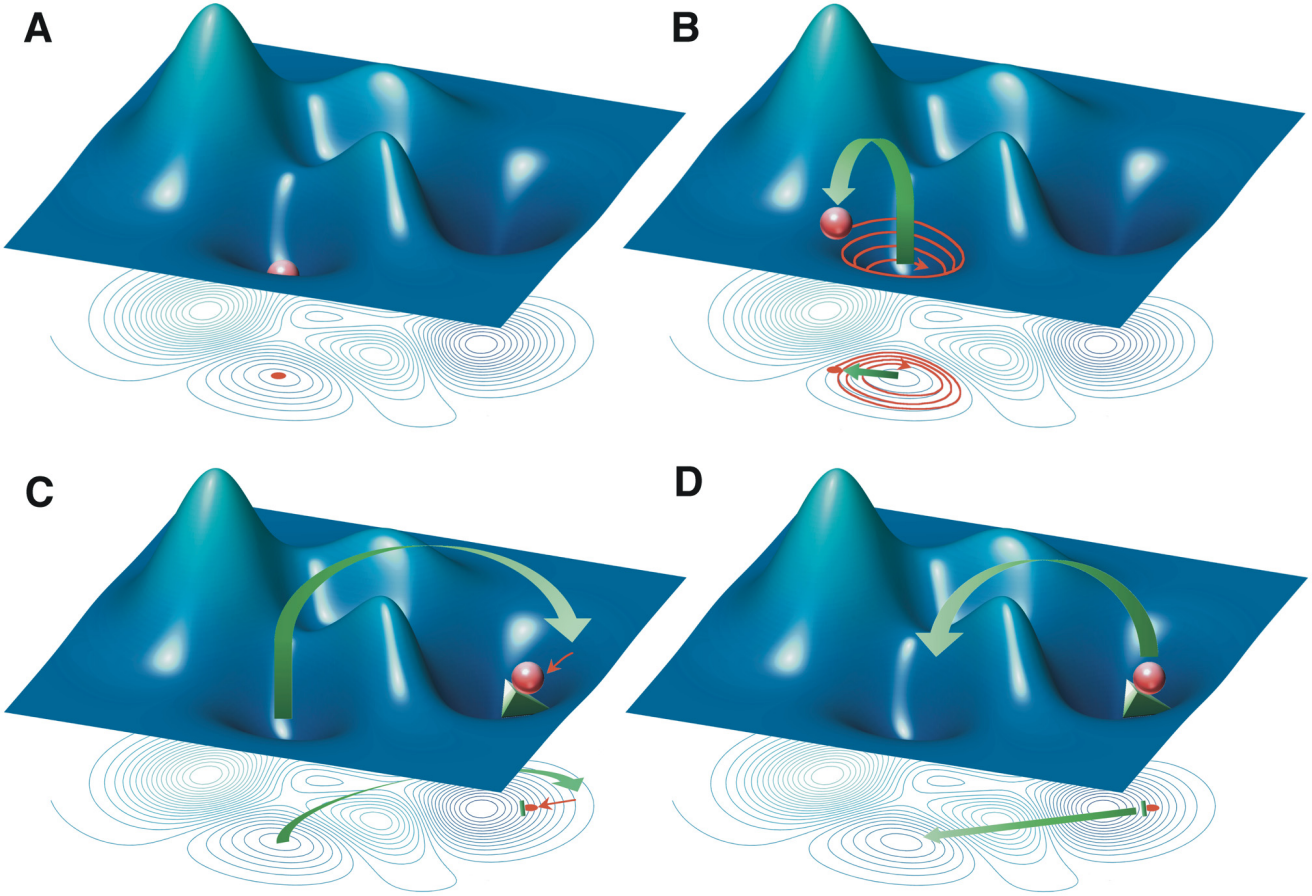
Illustration of homeostatic regulatory landscape. (A) The state of the system (red sphere) at rest in the typical homeostatic stable state. (B) Small external perturbation (green arrow) moves the system out of homeostasis, but dynamics return the system to its typical resting state (red arrow). (C) Large external perturbation moves the system into the basin of an alternate homeostatic stable state, but is held at a distance due to a continuing insult (green wedge). (D) Treatment course designed to move system back into the typical healthy basin of attraction. This image is a reproduction of the original found in [15] and presented under the Systems Biomedicine Creative Commons Attribution-NonCommercial 3.0 Unported License.

An intricate network of regulatory interactions, containing feed-forward and feedback loops like the HPA-HPG-immune axis, can produce a wide variety of response dynamics including multiple stable states that exist beyond normal homeostasis [15–20] (Fig 1A). Small perturbations to such a system result in a regulatory response that will return the system back to its normal homeostatic state (Fig 1B). However, under certain more extreme disturbances the system may be forced from its normal basin of regulation towards a new stable regulatory regime (Fig 1C). This disturbance may consist of a single isolated event alone, or may be compounded by a continuing external insult to the system that further alters the response dynamics (Fig 1C). Once the system has adopted an alternate basin of attraction, the corresponding regulatory dynamics now in effect will resist change in favor of the new homeostatic state even if such a state is chronic illness. We believe that these regulatory forces are at the very heart of resistance to therapy and may explain some of the difficulties in producing lasting remission in GWI. By characterizing these homeostatic regimes it may be possible to harness these regulatory forces and design minimally invasive treatment interventions that essentially move the system back into the regulatory pull of the healthy homeostatic basin (Fig 1D).

**Fig 2:**
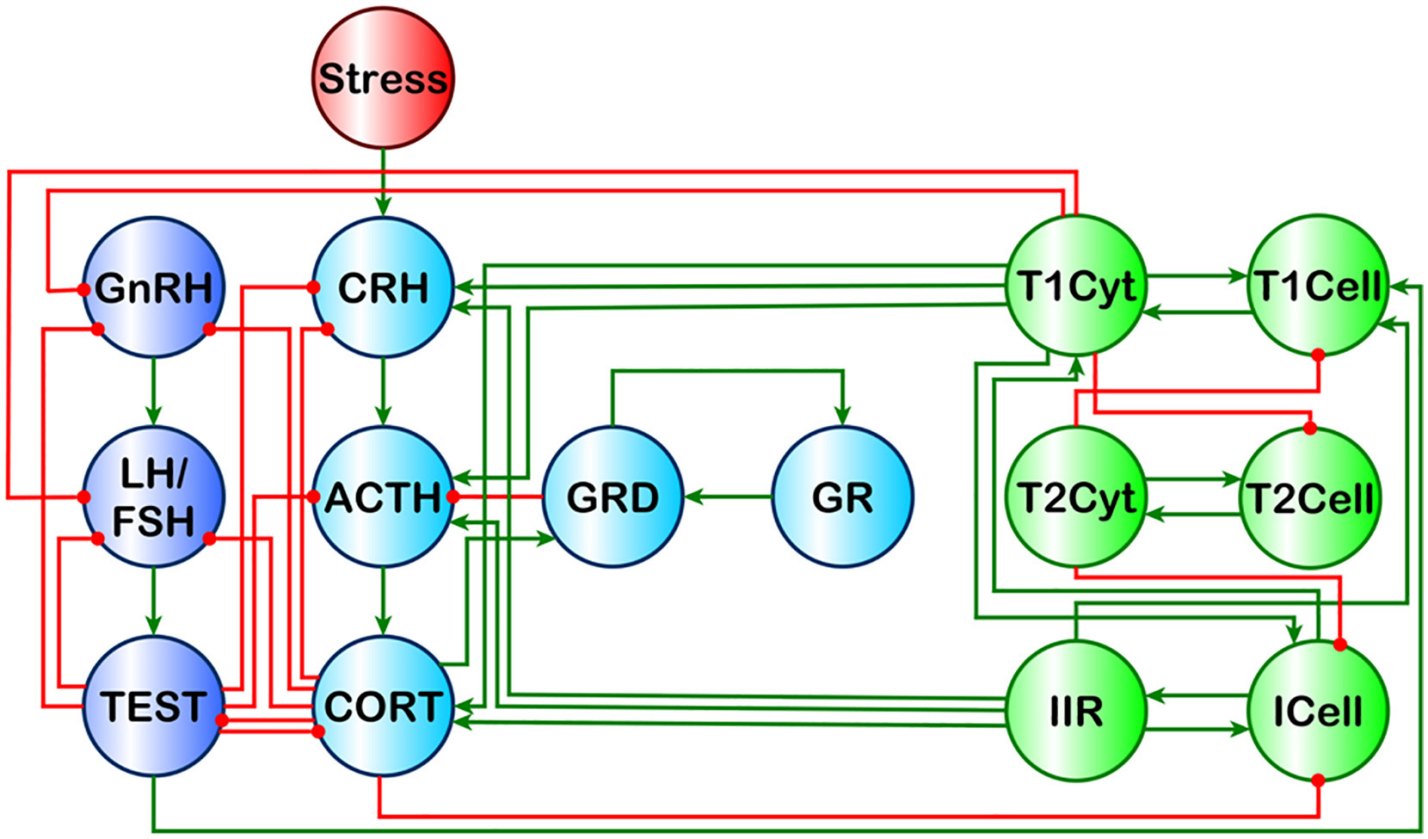
Theoretical Male HPA-HPG-Immune Signaling Network. Light blue nodes denote the HPA axis model described by Gupta et al., 2007 [14]. Dark blue nodes denote the male HPG axis. Green nodes denote a simplified immune system originally described in [16]. Red nodes denote external influences on the system. Green edges are stimulatory, and red edges are inhibitory. This image is a reproduction of the original found in [16] and presented under the PLoS ONE Creative Commons Attribution License.

In previous work we used a discrete logic model to show that multiple stable states exist for a simple HPA-HPG-Immune network [16]. Of these stable states the endocrine-immune profile observed experimentally in a GWI cohort aligned best with a stable state consisting of elevated cortisol and low testosterone with a shift towards Th1 immunity. This alignment does not imply that homeostatic drive is the root-cause of GWI, but rather that alignment with one of these multiple stable states might serve to sustain the chronicity of this illness. These alternate homeostatic regimes are by definition refractory and involvement of these forces could also promote resistance to therapy. Thus, these natural regulatory barriers to change must be considered in the design of any treatment avenue if robust remission from illness is to be achieved. Here we propose such a framework. Specifically we apply numerical modeling and simulation in a high-performance computing environment to identify potential treatments that guide regulatory equilibrium to a healthy homeostatic state. This is done by conducting Monte Carlo simulations on our previously reported discrete ternary logic model of combined HPA-HPG-Immune regulation in males (Fig 2) [16]. Using this methodology we first characterize the basin of attraction surrounding the illness homeostatic state by perturbing the system and allowing it to come to rest. In this way we define the closest common intermediate transitory states separating illness and health. Based on this we perform a global search using a Genetic Algorithm to find a sequence of externally induced state changes that direct the system to evolve along the most robust trajectory to normal homeostasis. In the case of GWI we show that a sequence of two interventions involving externally applied modulation of Th1 immunity followed by glucocorticoid receptor blockade is potentially capable of delivering sustained relief in a significant proportion of simulated GWI cases. Importantly the strategy presented is not illness specific but can be applied to a broad range of other complex chronic illnesses.

## Methods

### Male Model of Neuroendocrine-Immune Interaction

Current physiological and biochemical literature describes the HPA, HPG and immune systems as separate entities in great detail, but describes interactions across systems to a much lesser extent. In previous work we conducted a broad review of literature to develop a multi-system model incorporating these interactions linking the HPA, HPG and immune systems in males (Fig 2) and in females in order to determine available modes of homeostatic regulation [16]. Our analysis of the control logic governing these systems pointed to sexually dimorphic responses capable of supporting multiple homeostatic modes aligning with both normal health and pathologic dysfunction of the endocrine-immune axis. Experimentally measured biomarker profiles in blood from male GWI subjects showed a preferred alignment of GWI with one of these alternate attractor states characterized by hypercortisolism, low testosterone and a shift towards a Th1 immune response. This alternate homeostatic mode (AHM) resides within a basin of attraction that is separated from the healthy homeostatic mode (HHM), and reachable only via clinically deployed external perturbation of the system.

### Discrete Ternary Logical Analysis

The discrete ternary logical network analysis used in the present work is an extension of a methodology proposed by Mendoza and Xenarios [17] and Thomas [20], and has been reported previously by our group [15,16]. We encode documented feedback mechanisms within the endocrine-immune system using only the direction (source and target) and type (activator or inhibitor) of interaction. As data describing the magnitude of changes remains limited, we consider all cell types to be equally responsive to the actions of the cytokines for which they express receptors. Accordingly we also consider cytokine synthesis to be equivalent regardless of cell type. Using this formalism, we determine the number and type of stable resting states supported by the regulatory circuitry as well as the specific qualitative endocrine-immune signatures at each of these stable points without requiring detailed kinetic information. That is, we determine where the system would eventually come to rest even though we may not know how quickly this equilibrium will be reached.

In this model, signaling molecules and cell types are represented as individual variables each capable of adopting 3 discrete states: -1 (down-regulated), 0 (nominal), and 1 (up-regulated). At any point in time *t*, the state of a system with *N* variables can be represented by the vector $\vec{x}(t)$, such that:

$$\vec{x}(t)\;=\;(x_{1}(t),x_{2}(t),\ldots ,x_{N}(t))$$ (1)

where *x*_*i*_(*t*) is the state of the *i* ^*th*^ variable of the *N* variable system at time *t*. The image vector $\vec{x}(t\;+\;1)$ describes the preferred state towards which the system evolves in the next time increment. The state value of the image vector for the *i* ^*th*^ variable is determined from its current state and a set of balanced ternary logic statements based on the current value of variable and the mode of action (i.e. activate or inhibit) of the neighboring input variables. These logic statements are expressed as follows (Eq 2):

$$x_{i}(t+1)=\{\begin{array}{c} \left(x_{i1}^{A}(t)\vee x_{i2}^{A}(t)\vee \ldots \vee x_{iX}^{A}(t))\nabla (x_{i1}^{I}(t)\vee x_{i2}^{I}(t)\vee \ldots \vee x_{iY}^{I}(t)\right)\\ \left(x_{i1}^{A}(t)\vee x_{i2}^{A}(t)\vee \ldots \vee x_{iX}^{A}(t)\right)\\ \neg (x_{i1}^{I}(t)\vee x_{i2}^{I}(t)\vee \ldots \vee x_{iY}^{I}(t)) \end{array}$$ (2)

where the ∇, ∨, and ¬ symbols are ternary HIGH/LOW PASS, OR and NOT operators, $x_{ij}^{A}$ is the state of the *i* ^*th*^ variable’s *j* ^*th*^ activator, $x_{ik}^{I}$ is the state of the *i* ^*th*^ variable’s *k* ^*th*^ inhibitor. The ternary operators given in Eq 2 are described in further detail in [16]. The first entry in Eq 2 is used when the variable possesses *X* activators and *Y* inhibitors, the middle when the variable has only *X* activators and last when the activator has only *Y* inhibitors.

The number of nodes determines the total number of states available to a model, such that a model of *N* nodes possesses *3* ^*N*^ states. Due to this rule the number of total states increases rapidly as new nodes are added.

### Monte Carlo Simulation of State Evolution

**Fig 3:**
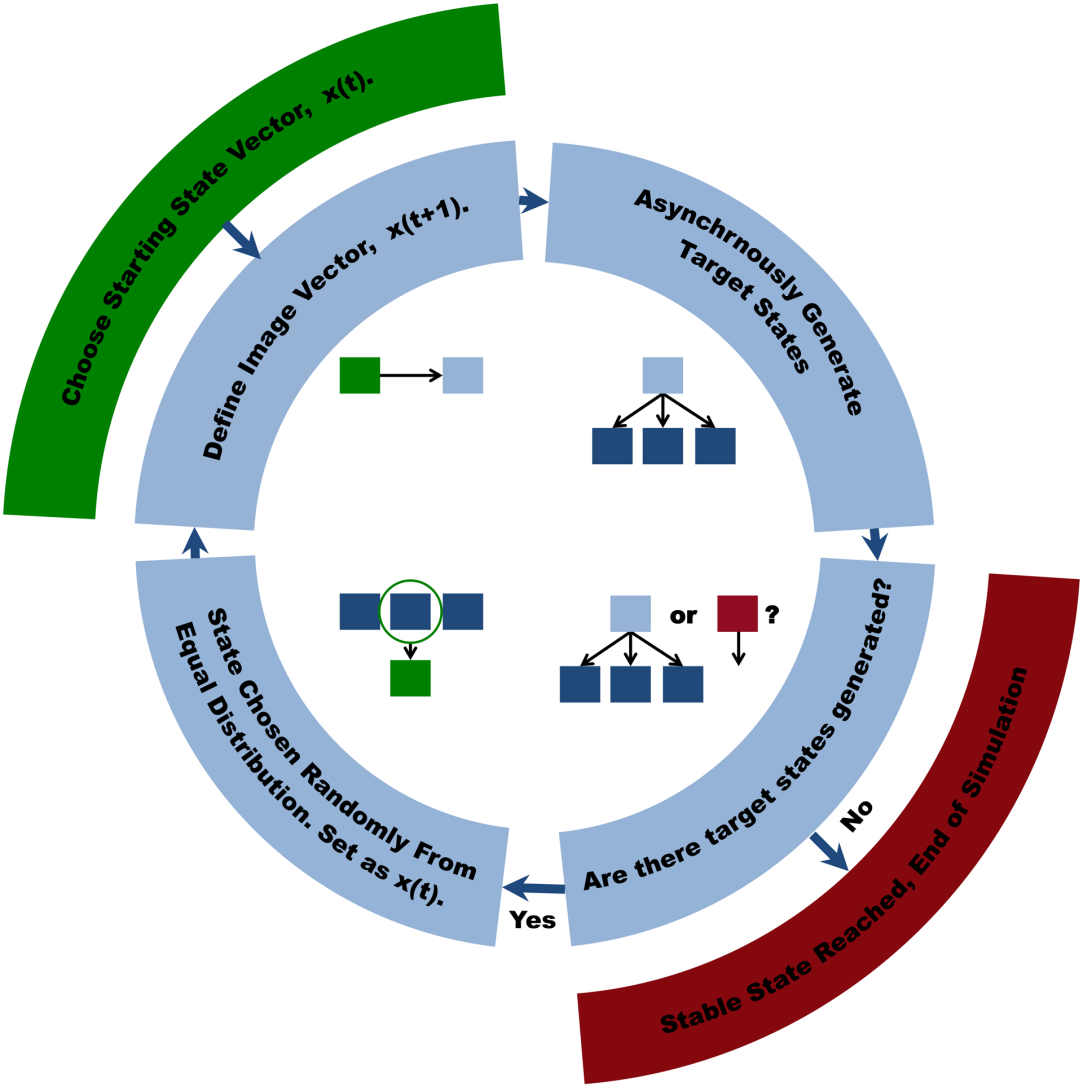
Monte Carlo Simulation Scheme for Analyzing the Evolution of the Discrete Ternary Logic Representations.

The evolution of state transitions supported by the model was analyzed by developing a Monte Carlo simulation algorithm. From any initial starting state, allowable state transitions are determined based on Eq 2. Applying Eq 2 to each variable in the model for the *m* ^*th*^ state of the system, ${\vec{x}}^{m}(t)$, defines the image vector ${\vec{x}}^{m}(t+1)$ for the *m* ^th^ state. With ${\vec{x}}^{m}(t+1)$ defined, the system may be updated asynchronously (allowing only one variable to change at a time) following the generalized logical analysis of Thomas [20]. According to this method the *i* ^*th*^ variable of the *m* ^*th*^ state vector $x_{i}^{m}(t)$ is moved one step towards its preferred image $x_{i}^{m}(t+1)$ (e.g. If $x_{i}^{m}(t)$ = -1 and $x_{i}^{m}(t+1)$ = 1, then $x_{i}^{m}(t+1)$ is set to 0). Thus, for each current state of the system there are potentially several subsequent states towards which it may asynchronously evolve. From the allowable transitions a target state is chosen at random using a uniform equal distribution and used to generate the next set of allowable target states. States for which the image vector is the same as the current state vector are considered stable (steady states, attractors, basins etc.), and do not evolve further in time. The Monte Carlo procedure is performed until such a stable state is reached. Executing the simulation multiple times gives a distribution of paths that is used to determine the behavior of the system from any given start state. This procedure is illustrated in Fig 3.

### Simulating Intervention Courses

To identify a robust sequence of interventions capable of moving the HPA-HPG-Immune system from a pathological mode of regulation to that of normal health we evolved solutions combining a specific choice of treatment targets as well as the sequence, spacing and type of external perturbation. For each of these candidate treatment courses, simulations were conducted to evaluate the occurrence of normal homeostasis. Specifically, each clinical intervention was represented as a treatment vector with N variables. Interventions applied to the system state at some point in time *t*, were represented by the vector $\vec{T}(t)$, such that:

$$\vec{T}(t)=(T_{1}(t),T_{2}(t),\ldots ,T_{N}(t))$$ (3)

where *T*_*i*_ is a ternary value describing the effect of the clinical treatment on the *i* ^*th*^ element of the system: -1 (suppressing), 0 (untreated), and 1 (elevating). At those time points where an intervention is being applied, the image vector $\vec{x}(t+1)$ describing the preferred state towards which the system should evolve is now defined as:

$$\vec{x}(t+1)=\vec{x}(t)+\vec{T}(t)$$ (4)

as opposed to the unperturbed logic described in Eq 2. Due to the ternary nature of this system no value can extend beyond the range of -1 to 1, hence values beyond this range were rounded accordingly (i.e. if *x*_*i*_ (*t*) = 1 and *T*_*i*_ (*t*) = 1, then *x*_*i*_ (*t* + 1) = *x*_*i*_ (*t*) + *T*_*i*_ (*t*) = 2 is rounded to 1). At times *t* when there is no treatment applied (i.e. all *T*_*i*_ = 0), state transition continues according the logic in Eq 2.

### Defining the Basin of Attraction Landscape

The AHM resides within a basin of attraction separated from the HHM. Characterization of the basin of attraction landscape was determined through the identification of the clinically relevant closest common ancestor states between the HHM and the AHM. Analyzing the landscape of the basin was accomplished by applying simulated treatments to the AHM at time *t* = 0 and then allowing the system to evolve naturally, according to the defined rules of logic, without further treatment intervention. These initial treatment perturbations were selected from a list of clinically feasible interventions involving multiple state variables. Here candidate interventions were allowed modulate ACTH, CORT, GR, IIR, Th1Cyt, Th2Cyt and TEST. This led to 3^7^ = 2187 initial treatment states. Each treatment was simulated 10,000 times.

Each simulation produces a path of state nodes that were visited during the course of execution. By repeating the same simulation many times we obtain a distribution of the resulting end states that are then used to determine the viability of a treatment course. Inspecting the path also gives us an idea of how difficult it might be to escape the pathological steady state. For each attempted treatment, we gauge this attractor landscape by recording the number of simulations successfully reaching the HHM versus those that fall into a non-HHM attractor. This is recorded as a percentage (% HHM) indicating the portion of the 10,000 simulations for that treatment course that reached healthy homeostasis.

### Genetic Algorithm for Optimizing Treatment Course

A first mapping of the illness basin of attraction consisted of a series of simulations where we first allowed for a one-time simultaneous perturbation of two or more variables at the outset only without subsequent intervention. We then expanded on this by performing a global search to find an optimal series of single target interventions separated in time, which reliably led to health. A Genetic Algorithm (GA) based search [18] was used to optimize this treatment course, as its form naturally accommodates the discrete definition of each system state.

A treatment course vector $\vec{C}$ with *M* interventions is therefore defined as,

$$\vec{C}=(\vec{T}(t_{1}),\vec{T}(t_{2}),\ldots ,\vec{T}(t_{M}))$$ (5)

where $\vec{T}(t_{i})$ is the intervention treatment vector at the *i* ^th^ intervention time point. Due to the asynchronous nature of the model each treatment vector $\vec{T}$ only contains a single target intervention *T*_*i*_ that affects the *i* ^th^ element variable in the system at any given time step.

**Fig 4:**
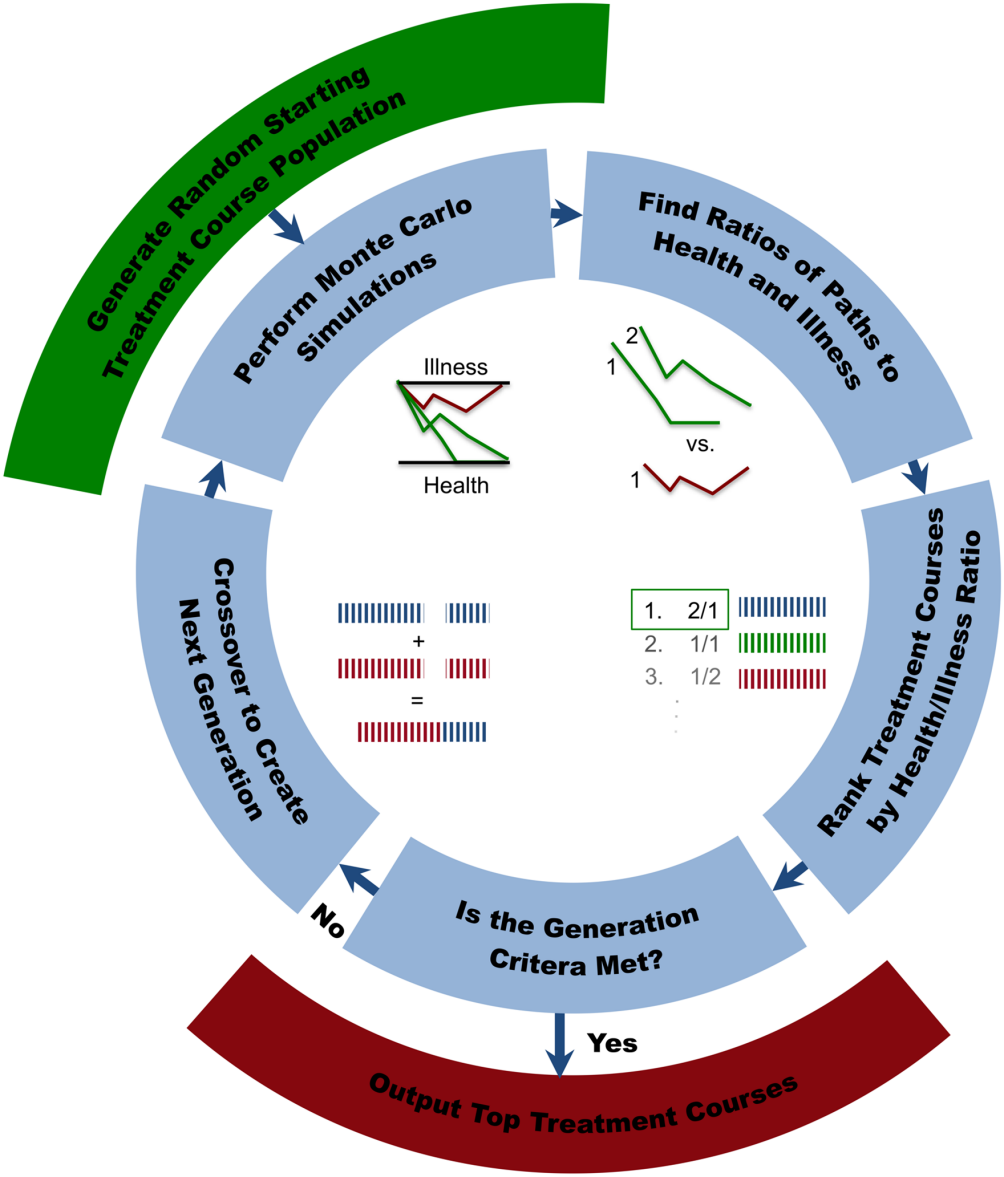
Scheme of Genetic Algorithm Optimization of Treatment Course.

The GA (Fig 4) starts by generating a population of 1000 candidate treatment courses each composed of a specific number of randomly selected interventions applied at random time points. The response of the system to each treatment course in this initial generation of candidates is then simulated for 1000 time steps. Over the course of these time steps the state of the system evolves according to Eq 2, except at those times when interventions are applied. At these intervention events the state transition follows Eq 4. These 1000 iterations provide a distribution of paths that are then ranked according to a fitness function based on the number of times a treatment successfully reaches the healthy stable state (% HHM). After all treatments in the generation have been ranked, the top 10% are retained without change for the next generation. Thus, treatments generated by the GA that have a higher probability of leading to the HHM are re-executed and re-evaluated over many generations. The remainder of the next generation of candidate solutions is created by choosing random pairs from the total set of treatment courses (including the top 10^th^ percentile) and combining (cross-over recombination) them. Combination, ⊗, of two treatment courses $\vec{C_{1}}$, and $\vec{C_{2}}$ is performed at a single random splice point *s* to create a new treatment course ${\vec{C_{1}}}^{\prime }$ for the next generation, such that,

$$\begin{array}{c} \vec{C_{1}}⊗\vec{C_{2}}\Rightarrow {\vec{C_{1}}}^{\prime }\\ \left(\vec{T_{1}}(t_{1}),\ldots ,\vec{T_{1}}(t_{s})\left|\vec{T_{1}}(t_{s+1}),\ldots ,\vec{T_{1}}(t_{M}))⊗(\vec{T_{2}}(t_{1}),\ldots ,\vec{T_{2}}(t_{s})\right|\vec{T_{2}}(t_{s+1}),\ldots ,\vec{T_{2}}(t_{M})\right)\\ \Rightarrow (\vec{T_{1}}(t_{1}),\ldots ,\vec{T_{1}}(t_{s})|\vec{T_{2}}(t_{s+1}),\ldots ,\vec{T_{2}}(t_{M})) \end{array}$$ (6)

The response to the new treatment courses, including members of the previous top 10^th^ percentile, are then simulated once again and ranked as a new generation. This process is continued iteratively for 1000 generations. The final treatment course with the highest % HHM was taken as the best treatment solution for a given run. The overall best treatment course was chosen from 100 repeated GA runs.

## Results

### Response to Single Intervention Event

**Fig 5:**
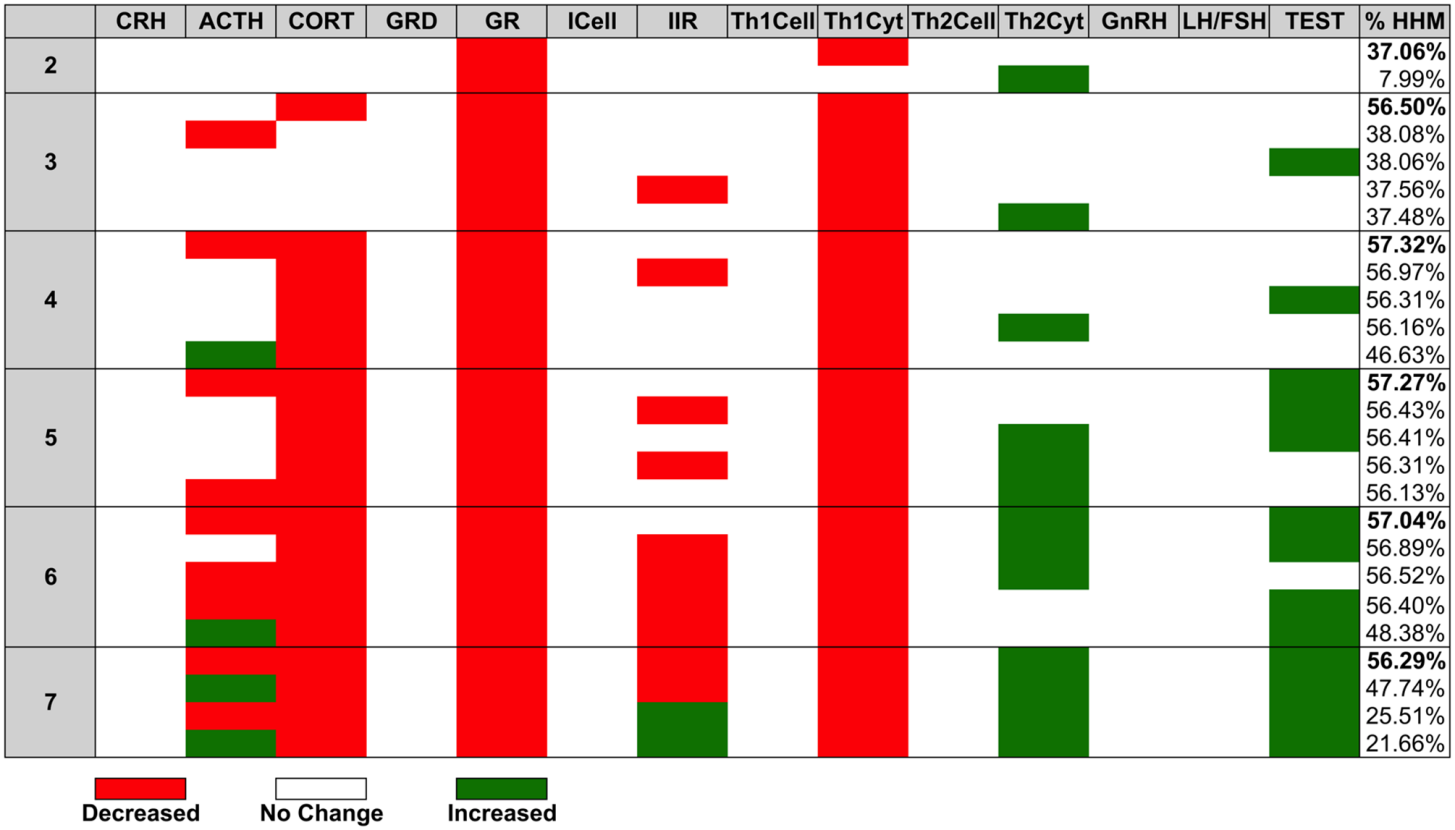
Depth of the GWI basin of Attraction.

From our previous investigation of normal HPA-HPG-Immune regulation in male subjects (Fig 2) we found that the endocrine-immune profile experimentally measured in male GWI subjects most closely aligned with an AHM characterized by hypercortisolism, low levels of testosterone and a shift towards Th1 immune activation. The landscape of these basins of attraction was determined through the identification of the clinically relevant closest common ancestors between the HHM and the AHM. Results for these simulations showing the percentage of runs returning to the HHM are found in Fig 5. We found that no single intervention event allowed for a robust return from the AHM to the HHM indicating that two or more interventions are required. The least invasive treatment in the set of healthy outcomes included two of the treatable state variable nodes as targets. The best of these two-node treatments involved inhibiting GR and Th1Cyt. This two-target single intervention event produced a healthy outcome ~37% of the time in the 10,000 simulations. The single intervention treatment with the highest probability of delivering lasting remission involved four targets. To deliver a lasting remission with a maximum probability of ~57% required inhibiting ACTH, CORT, GR, and Th1Cyt.

All single intervention courses resulting in a lasting return to HHM involved inhibition of GR, and all but one involved inhibition of Th1Cyt. This suggests that these two targets may constitute core treatment targets in more elaborate multiple intervention courses. The addition of additional interventions improved the overall probability of return to HHM however at the cost of invasiveness.

### Optimizing the Delivery of Multiple Intervention Events

Analysis of the AHM basin of attraction (above) indicates that multiple interventions are necessary to move the system to the HHM. As our discrete logic the system may be updated asynchronously, we use a genetic algorithm coupled to our simulation to optimize the number, timing and type of interventions in a treatment course to move the system from the AHM to the HHM. Simulations were executed for treatment courses composed of 2 to 8 separate intervention events applied across multiple targets. Again, the target state variables defined as available for intervention were ACTH, CORT, GR, IIR, Th1Cyt, Th2Cyt and TEST.

**Fig 6:**
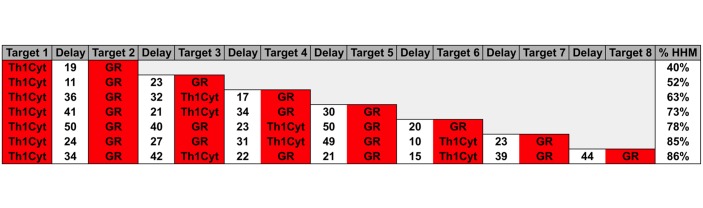
GA Simulation results.

The simulated treatment courses that resulted in the highest fitness values or the highest percentage of endpoints corresponding to a stable remission are listed in Fig 6. Without exception all interventions involved an initial inhibition of Th1 inflammatory cytokines (Th1Cyt) followed by a subsequent inhibition of glucocorticoid receptor function (GR). These first two intervention events alone ended in stable and lasting return to the HHM in 40% of the simulated cases. Subsequent interventions consisted of additional treatment cycles alternating between repeated inhibition of Th1Cyt and/or GR. For example a second cycle of Th1Cyt and GR blockade produced a predicted remission rate of roughly 2 out of 3 simulated subjects (63%). With 4 cycles (8 treatment interventions) of alternating Th1Cyt/ GR blockade separated by intermediate repeated suppression of GR delivered a maximum expected remission of rate of 86%. The intervals between intervention events varied between 10 and 50 time steps and depended on the specific sequence of treatment.

### Simulating an Optimized Treatment Course

**Fig 7:**
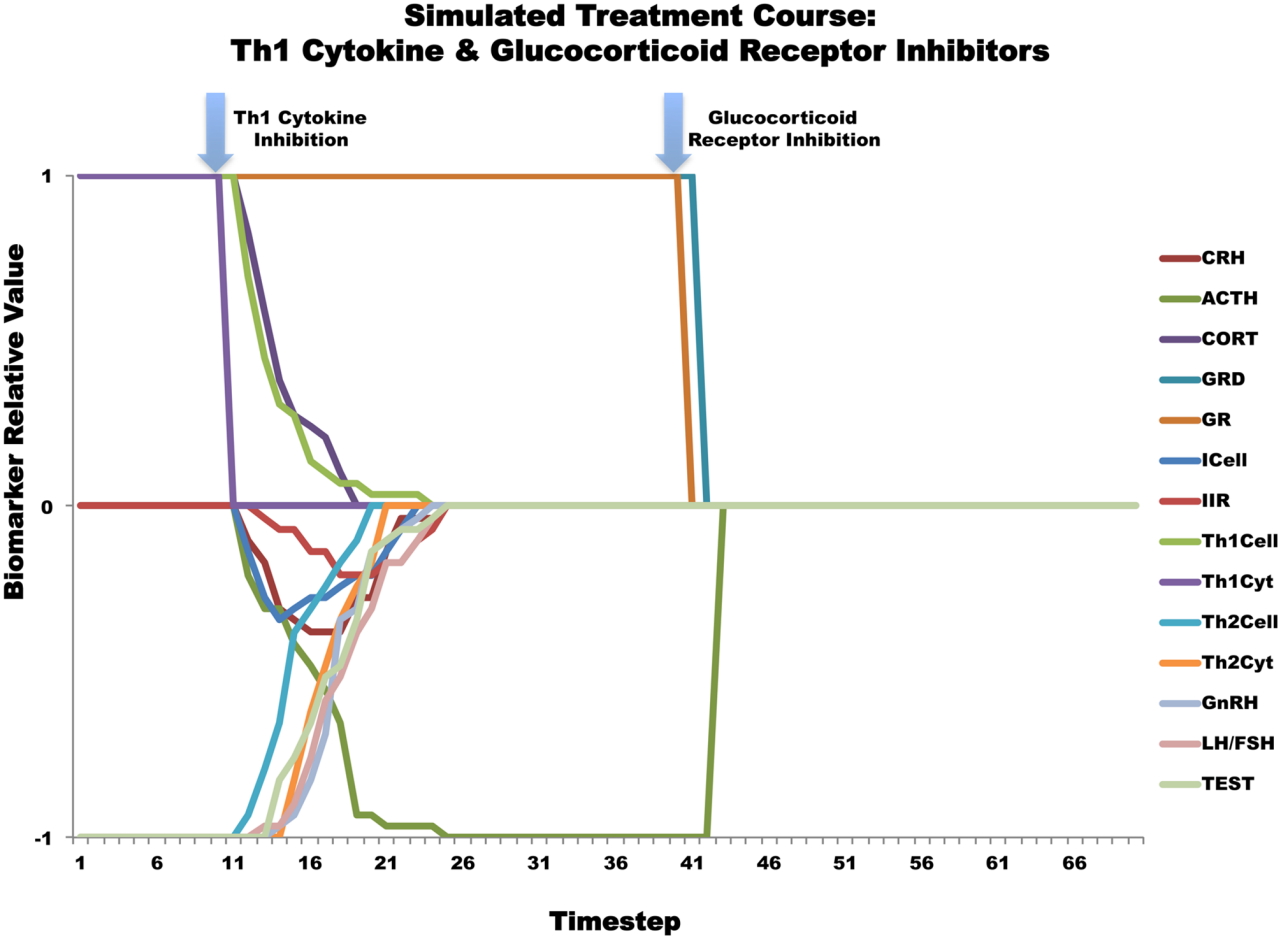
Average values of simulated Th1Cyt—GR inhibition treatment course.

Due to the limited availability of detailed kinetic data, the models used in this work strictly enforce the direction and sequence of regulatory control actions but do not represent the response time. Although the intervals described in Figs 6 and 7 are reported as simulation time steps, the actual timescale of these treatments courses can nonetheless be extracted in the form of landmark states. To provide a more robust approximation of the rate of success and to glimpse the intermediate transitory states occupied by the system, a minimally invasive single treatment cycle of Th1Cyt/ GR inhibition was simulated 1000 times. The value of each node variable was averaged for each time-point and plotted as a function of the time-step for all simulations leading to the HHM (Fig 7). These simulation results indicate that Th1Cyt inhibition alone causes the system to move from the GWI illness state characterized by high CORT, low TEST and a Th1 shift towards one of the other alternate homeostatic modes (AHM) predicted in our previous work [16], with high GRD, GR and low ACTH. Treatment of this second alternate mode with inhibition of GR pushes the system towards normal homeostasis. Importantly these simulations suggest that the second intervention involving GR blockade should not be applied until ACTH levels reach a stable minimum. Only then should a GR blockade be attempted. As HPA dynamics may be expected to vary from one individual to the next, these intervals will fluctuate and such landmark states might in reality prove to be more useful than a standardized time period. Simulations also showed that subsequent cycles of Th1Cyt and GR inhibition produce similar responses and serve to redirect those trajectories that did not respond to the first two-step treatment course, thus increasing the overall percentage of trajectories reaching health.

## Discussion

In previous work we presented discrete ternary logic models of immune system interaction with stress and sex hormone regulation [15,16]. We found that these regulatory networks naturally supported more than one stable homeostatic regime and that immune and endocrine signatures observed in GWI aligned much more readily with an altered regulatory regime characterized by Th1 activation, elevated cortisol and depressed testosterone. In the present work we investigated putative treatment courses aimed at guiding this dysregulated HPA-HPG-Immune system back to healthy homeostasis, specifically for the case of GWI. Therapeutic avenues for GWI were first identified by characterizing the basin of attraction surrounding the GWI alternate homeostatic state. By defining the closest common transitory states shared between illness and health our simulations indicated that that no single treatment target supports a return to normal regulation and that at least two separate interventions are needed: namely inhibition of glucocorticoid receptors (GR), and Th1 cytokines (IL-2, IFN-γ, TNF-β). Using Genetic Algorithm to search for an intervention strategy and dosing schedule that maximizes the predicted remission rate we found that a treatment course consisting of repeated cycles of Th1 cytokine inhibition followed by glucocorticoid receptor blockade results in the best overall chance of restoring normal homeostatic control of the HPA-HPG-Immune system in GWI.

Elevated Th1 immune responses, in the context of varying Th2 and Th17 signals, have been implicated in multiple human disease states. For example, in rheumatoid arthritis TNF-β levels in the synovium are elevated [21], as are serum levels of IL-2 [22]. High serum IL-2 levels are also associated with conditions such as scleroderma, progression of gastric and non-cell lung cancer, and certain neoplastic diseases [22], while elevated IFN-γ is implicated in pathology of diseases such as systemic lupus erythematosus [23], and multiple sclerosis [24]. Consistent with our models, experimental data reported previously by this group [25] indicate that GWI subjects often present a loss of Th1:Th2 polarity as evidenced for example by concurrently by significantly elevated IL-13 (Th2) and TNF-β (Th1). Though our data shows a broad range of values for IFN-γ levels in peripheral blood from GWI subjects [25], this central Th1 cytokine also tends to be over-expressed in this illness group. Skowera et al. previously reported significantly elevated levels of IL-2, IFN-γ and IL-4 producing CD4+ cells for GWI in non-stimulated culture compared with asymptomatic veterans [26]. Zhang et al. found significantly higher levels of mRNA for a number of Th-1 markers such as IFN-γ, TNF-α, IL-2 and IL-10 in Gulf War veterans diagnosed with chronic fatigue syndrome (CFS) compared to CFS civilian controls [27]. Allen et al. found an approximately equal mix of Th-1 (IFNγ and IL-2) and Th-2 (predominantly IL-13) recall response to anthrax vaccine, whereas response to plague was polarized toward Th-1 in male GW veterans [28]. This evidence supports the existence of elevated Th1 cytokines in veterans with GWI, at rest, however, it must be noted that while GWI immune networks portray a Th1 motif at rest, under stress these networks can shift to a Th2 profile [29]. Consistent with our previous modeling efforts [15,16], this suggests that immunological profiles of Gulf War illness may be state specific, only becoming apparent under “challenge” conditions that exceed an individual’s capacity for homeostatic compensation. As the optimization presented here is directed at restoring normal homeostasis instead of transitory response it may not be unexpected to find a focus on remediation of Th1 activity as a key component of the prescribed treatment course.

While it appears that a Th1 imbalance is a component in GWI, to our knowledge there have been no studies examining Th1 inhibition in GWI. Certain autoimmune conditions, specifically multiple sclerosis and uveitis that involve inflammation of neurological tissue, have shown responsiveness to anti-IL2Rα antibodies [30–34]. Two anti-IL2Rα antibodies commonly used in transplant rejection therapy, daclizumab and basiliximab, have undergone phase II trials for MS and uveitis, however neither of them are currently on the market [30]. Anti-IFN-g treatment also appears to improve Th1 autoimmune diseases, including MS and RA [35]. Specifically, randomized studies of antibodies to IFN-g showed statistically significant improvement in disability progression in secondary progressive MS [36], and improved symptoms in RA [37]. To our knowledge, no trials of anti-IL-2 or anti-IFNg in the treatment of GWI are currently under investigation.

Hypercortisolism, excess levels of the stress hormone cortisol, has been observed in patients suffering from a number of conditions including the muscle wasting condition Cushing’s syndrome [38,39], the memory deteriorating Alzheimer’s disease [40,41], and chronic pain [42,43]. Experimental data reported previously by this group [25] indicate that GWI subjects tend to present with chronically elevated levels of CORT. Glucocorticoid receptor antagonists, which block the effects of cortisol, are currently in use to both diagnosis and treat disorders associated with elevated cortisol levels [44,45]. Mifepristone, an FDA approved powerful glucocorticoid receptor antagonist, is used in the treatment of Cushing's syndrome [46,47]. Its efficacy has been studied in a clinical trial of a small cohort of military veterans with combat related post-traumatic stress disorder (PTSD) [48], finding that mifepristone treatment produced clinical improvement in PTSD symptoms. According to Nicolson et al., veterans suffering from GWI are often diagnosed with PTSD yet evidence linking PTSD and GWI is based on the assumption that veterans must have suffered from some form of battlefield stress while in the Gulf War theater [49]. Indeed veterans with GWI have been shown to have significantly higher rates of PTSD, supporting the idea that GWI symptoms are exacerbated by stress. A recent population-based study indicates that 35% of veterans with GWI were estimated to have PTSD [50]. However, the overall effects of past traumatic events on the clinical presentation of GWI have not been evaluated. Clinical trials of mifepristone treatment in Gulf War veterans with chronic multi-symptom illness (i.e. GWI) are currently underway [51]. Our results suggest that glucocorticoid inhibition alone will not result in a return to healthy neuro-endocrine immune regulation.

Our simulations predict that timed treatments targeting Th1 cytokines followed by gluccocorticoid receptor activity will provide the highest chances for moving the system from an elevated cortisol, low testosterone, increased Th1 activation state towards healthy behavior. However, it must be noted that results presented here were derived from simulations based on an idealized model and that the granularity and accuracy will be dictated accordingly. Currently this model does not account for detailed kinetics, as data describing the magnitude and transition time of interactions between elements of the extended neuroendocrine-immune system are not available. Refinement of this model by parameters obtained from data driven analysis will serve to improve simulations and reliability of results. Ultimately, even with these refinements, safety and efficacy of these predicted strategies must be determined clinically.

While our focus here has been on GWI, the regulatory model is not specific to GWI pathology and the methodology presented is fully generalizable to other complex illnesses. We have shown a common multiple intervention strategy is capable of moving this complex multi-axis regulatory system from a persistent state of chronically elevated cortisol, low testosterone, and increased Th1 activation back into a robust homeostasis and normal endocrine-immune balance.
